# Content Representation of Tactile Mental Imagery in Primary Somatosensory Cortex

**DOI:** 10.1523/ENEURO.0408-22.2023

**Published:** 2023-06-02

**Authors:** Till Nierhaus, Sara Wesolek, Daniel Pach, Claudia M. Witt, Felix Blankenburg, Timo T. Schmidt

**Affiliations:** 1 Neurocomputation and Neuroimaging Unit, Department of Education and Psychology, Freie Universität Berlin, 14195 Berlin, Germany; 2 Charité – Universitätsmedizin Berlin, corporate member of Freie Universität Berlin and Humboldt-Universität zu Berlin, Institute of Social Medicine, Epidemiology and Health Economics, Charitéplatz 1, 10117 Berlin, Germany; 3 Institute for Complementary and Integrative Medicine, University Hospital Zurich, 8091 Zurich, Switzerland

**Keywords:** attention, fMRI, mental imagery, MVPA, S1, vibrotactile stimulation

## Abstract

The imagination of tactile stimulation has been shown to activate primary somatosensory cortex (S1) with a somatotopic specificity akin to that seen during the perception of tactile stimuli. Using fMRI and multivariate pattern analysis, we investigate whether this recruitment of sensory regions also reflects content-specific activation (i.e., whether the activation in S1 is specific to the mental content participants imagined). To this end, healthy volunteers (*n* = 21) either perceived or imagined three types of vibrotactile stimuli (mental content) while fMRI data were acquired. Independent of the content, during tactile mental imagery we found activation of frontoparietal regions, supplemented with activation in the contralateral BA2 subregion of S1, replicating previous reports. While the imagery of the three different stimuli did not reveal univariate activation differences, using multivariate pattern classification, we were able to decode the imagined stimulus type from BA2. Moreover, cross-classification revealed that tactile imagery elicits activation patterns similar to those evoked by the perception of the respective stimuli. These findings promote the idea that mental tactile imagery involves the recruitment of content-specific activation patterns in sensory cortices, namely in S1.

## Significance Statement

It has been shown previously that mental imagery of sensations in different modalities can activate respective primary sensory cortices (visual/auditory/tactile). However, a relation of such activation to the imagined mental content was mainly shown for the visual system. Here, we generalize this concept to the somatosensory domain by showing that content-specific activation during tactile mental imagery can be found in primary somatosensory cortex subarea BA2. Most importantly, we show that tactile imagery elicits activation patterns similar to those evoked by sensory stimulation. Our results provide further evidence that sensory recruitment is among the brain processes that allow conscious information representation.

## Introduction

It is a fundamental cognitive ability of humans to mentally imagine diverse content in varying degrees of vividness. The underlying neural mechanisms that generate and process mental content in the absence of sensory stimulation remain subject to ongoing investigations, despite a long research history (Kosslyn et al., 2001; Tong, 2013; Nanay, 2018). Generally, frontal areas, such as the inferior frontal gyrus (IFG), medial frontal gyrus, and supplementary motor area (SMA), as well as multimodal parietal regions including the inferior parietal lobule (IPL) are most commonly reported to be activated during imagery across modalities (Hassabis and Maguire, 2009; Zvyagintsev et al., 2013; Schmidt and Blankenburg, 2019). These higher-order frontoparietal areas are thought to relate to the general mental construction process (e.g., by contributing attentional resources; Lückmann et al., 2014). However, these brain regions do not seem to code information about the mental content per se (Riggall and Postle, 2012). What types of neural activation reflect specific mental content is an open question in current research (Christophel et al., 2017).

With regard to this question, it has previously been shown that the imagery of a specific sensation, such as the imagery of being touched or seeing an object, can activate corresponding sensory regions (Kosslyn et al., 1995), a process referred to as sensory recruitment (Pasternak and Greenlee, 2005). Especially for the visual domain this has been frequently shown (Kosslyn et al., 1993; Klein et al., 2000; Kosslyn and Thompson, 2003; Ganis et al., 2004), while the activation of corresponding sensory cortices induced by auditory (Zatorre et al., 1996) or tactile mental imagery (Yoo et al., 2003; Schmidt and Blankenburg, 2019) have been examined less often. Sensory recruitment of the primary somatosensory cortex (S1) and the secondary somatosensory cortex (S2) was initially indicated by means of signal increases related to the imagery of brushing stimuli (Yoo et al., 2003). Furthermore, a somatotopic activation of the hierarchically highest subarea BA2 of S1 has been demonstrated during imagery of vibrotactile stimuli at different body parts (Schmidt and Blankenburg, 2019), and even down to BA1 during more detailed vibrotactile mental representations (Schmidt et al., 2014). The extent to which these activations in sensory regions directly relate to the content of mental imagery, however, remains an open question.

Multivariate pattern analysis (MVPA) has proven to be a useful approach for determining the content-specific activation of perception and mental imagery. Several studies have revealed shared neural codes for visual stimulation and corresponding imagery (Stokes et al., 2009, 2011; Reddy et al., 2010; Cichy et al., 2012). While Reddy et al. (2010) found the visual presentation of simple objects and their imagery to activate shared codes in the ventral temporal cortex, other studies revealed that imagery of simple shapes (distinct letter stimuli) activates the same neural representations within high-level visual cortex that are activated by corresponding visual stimulation (Stokes et al., 2009, 2011). But also early visual areas were shown to contain the content of mental imagery. Cichy et al. (2012) revealed that, in addition to category-selective regions and higher-order visual areas, also hierarchically lower subregions of the visual cortex (V1, V2, V3) contain shared neural codes of visual stimulation and imagery. And by applying MVPA to fMRI data, Albers et al. (2013) again showed that early visual cortices exhibit content-specific activation patterns during mental imagery. These studies in the visual system demonstrate that MVPA allows identifying content-specific activation not detectable by univariate analyses. With regard to the somatosensory system, it still needs to be tested whether content representation of tactile mental imagery can be found in S1.

In this fMRI study, we used three different types of vibrotactile stimulation (Press, Flutter, and Vibration) in a 2 × 3 factorial design [Stimulation (Stim) vs Imagery (Imag)] to test whether content-specific activity of mental imagery can be found in S1 by applying univariate and multivariate analyses. The stimuli were chosen so as to activate different mechanoreceptors in the skin, which are known to preferably respond to different features of tactile stimulation, such as pressure or vibration (Delhaye et al., 2018), and therefore should induce different patterns of somatosensory cortical activation. As the higher-order subregion BA2 of S1 was recently shown to be involved in mental tactile imagery (Schmidt and Blankenburg, 2019), we hypothesized that it is more likely to detect content-specific codes in BA2 than in the other subregions BA3b and BA1.

## Materials and Methods

### Participants

The final sample of participants included in the analysis comprised *N* = 21 participants (16 female, 4 male, 1 diverse; mean age ± SD, 24.90 ± 5.24 years). Twenty-seven participants completed the fMRI measurement. Of these, six participants were excluded because of low ratings on successful mental imagery (see below). All volunteers gave written informed consent to participate in the experiment and received remuneration for their time. Handedness of all participants was assessed using the Edinburgh Handedness Inventory (Veale, 2014). Within a range of −100 to +100 for fully left and right handedness, 20 of the included subjects were classified as right handed (mean ± SD laterality score, 77.8 ± 30.6), and one classified as left handed (laterality score, −23.1). All procedures were in line with the Declaration of Helsinki, and the study was approved by the local ethics committee.

### Procedure

After informing the participants about the experiment and safety requirements as well as checking their suitability for MRI, participants were instructed about the mental imagery task outside of the scanner and then familiarized with the task during the anatomic scan. Participants performed the task during six functional MRI runs interspersed with breaks on demand overall for ∼1 h. After leaving the scanner, they filled out the Vividness of Tactile Imagery Questionnaire (VTIQ; see below), concerning their overall ability to imagine tactile stimuli.

### Stimuli

**Figure 1.**
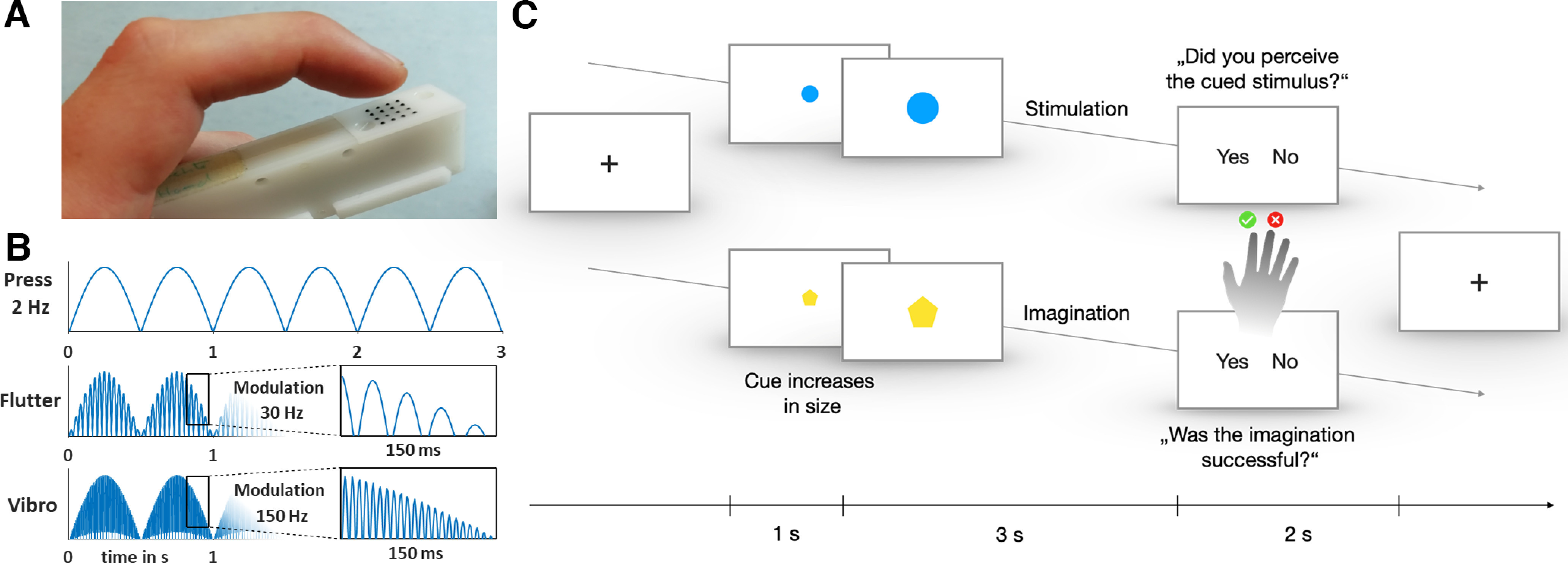
Design of the stimuli and experimental trials. ***A***, Piezoelectric Braille-like stimulation device. ***B***, Stimulation profile of the three conditions: Press (unmodulated 2 Hz half-sine wave pulses), Flutter (30 Hz modulated), and Vibro (150 Hz modulated). A stimulation always lasts 3 s. ***C***, Experimental design: one trial includes 1 s precue and three seconds of Stimulation or Imagery condition, followed by a response. In 25% of the Stimulation trials, no stimulation was applied. A fixation cross was presented during the variable intertrial interval. Note that the color and shape of the cues associated with conditions varied from participant to participant and was selected arbitrarily for this demonstration.

As applied in previous studies on mental imagery and perception (Schmidt et al., 2014, 2017; Nierhaus et al., 2015; Schröder et al., 2019, 2021; Forschack et al., 2020), the vibrotactile stimuli were presented to the left index finger via a piezoelectric Braille-like display (Piezostimulator, QuaeroSys, St. Johann, Germany) with 16 pins (4 × 4 matrix with 2.5 mm spacing; Fig. 1*A*). To ensure that the module was held in the same position throughout the experiment, it was taped to the participants’ index fingers. The three different stimulus types (Press, Flutter, and Vibration) were presented or had to be imagined for 3 s duration to allow clear perception and imagination. To optimally apply a slowly changing stimulus in the low-frequency range, the Press stimulus comprised 2 Hz half-sine wave pulses. To further minimize the physical and perceptual differences among the three stimulus types, for the other two stimulus types the 2 Hz half-sine wave pulses were modulated by 30 Hz (Flutt) or 150 Hz (Vibro), corresponding to the flutter and vibration frequency ranges, respectively (Fig. 1*B*).

Together with the vibrotactile stimuli, visual cues were presented, projected on a screen visible from within the scanner. Visual cues comprised three centrally presented shapes (star, circle, and pentagon) depicted in either blue or yellow. The color corresponded to the Stimulation or Imagery condition, and shapes indicated the Vibration type. Color and shape associations with stimulus type were balanced across participants.

### Experimental paradigm

The experimental paradigm comprised a 2 × 3 design, with the factors Stimulation/Imagery and stimulus types Press/Flutt/Vibro. Figure 1*C* illustrates the time course of one trial. Each trial started with a 1 s visual precue to prepare for the task. Then the cue increased in size to be shown for 3 s while the participants either perceived a stimulation or had to imagine it. In a quarter of the Stimulation trials, no stimulation was presented despite the presentation of a stimulation cue, and the participants had to attend to correctly respond whether or not a stimulus was presented. This trial design was chosen to assure that participants directed their spatial attention during all precues to the stimulation site. Finally, participants reported by a right-hand index/middle finger button response whether they felt a stimulation during the Stimulation trials or whether or not their imagination was successful. Left/right–yes/no assignment was balanced across trials. During the 2-, 3-, 4-, 5-, or 6-s-long intertrial intervals (ITIs), a fixation cross was presented. Each experimental run comprised 48 trials presented in a random order: six trials per experimental condition (*StimPress*, *StimFlutt*, *StimVibro*, *ImagPress*, *ImagFlutt*, *ImagVibro*), complemented with six No-Stimulation trials (two per stimulus cue) and six unannounced Null events occurring as a longer ITI in which the fixation cross was presented 6 s longer. Six runs of 8 min were conducted.

### Questionnaire assessment

Inspired by the Vividness of Visual Imagery Questionnaire (VVIQ) and its revised version (VVIQ-2; Marks, 1973, 1995), we developed the VTIQ to assess the participant’s ability for tactile imagery. As with the VVIQ, the VTIQ contains 16 items to determine the lifelikeness of an imagined sensation (see extended data for full questionnaire). The participants were asked to rate how precise, detailed, and similar to an actual sensation the imagery of different daily situations was. Responses were given on a 5-point scale. As in the VVIQ-2, high scores indicated a high lifelikeness of the mental imagery (1 = I don’t have any imagery, I only know that I’m thinking about the sensation described; 2 = Vague and blurry; 3 = Somewhat precise and vivid; 4 = Precise and vivid; 5 = Very precise and vivid, It feels like I’m actually perceiving the sensation described).

### FMRI data acquisition and preprocessing

The data were acquired on a 3 T MRI scanner (TIM Trio, Siemens) equipped with a 32-channel head coil. The whole brain was scanned in 37 slices of ascending order. A run comprised 242 functional T2*-weighted images (TR = 2000 ms; TE = 30 ms; matrix size = 64 × 64; flip angle = 70°; 3 × 3 × 3 mm^3^). Additionally, a T1-weighted image was acquired, comprising 176 sagittal slices (TR = 1900 ms; TE = 2.52 ms; 1 × 1 × 1 mm^3^).

Data preprocessing was mainly done using SPM12 (Wellcome Trust Centre for Neuroimaging, Institute for Neurology, University College London, London, UK) running on MATLAB, version 2021b (MathWorks). Additionally, the toolboxes Rest (version 1.8; Song et al., 2011) and dPABI (Yan et al., 2016) were used. To quality check the data and correct for potential compromised slices, the ArtRepair-Toolbox for SPM (Mazaika et al., 2005) was utilized and run with a default threshold of 18, which refers to the degree of accepted noise per scan. Only for six participants an interpolation of slices was suggested and realized as implemented in the ArtRepair-toolbox. For each of them, ≤5% of slices were identified for interpolation, together speaking for an overall low amount of motion in the data. The further preprocessing comprised slice time correction; realignment; coregistration; normalization to MNI space using unified segmentation, including reslicing of the functional data to 2 × 2 × 2 mm^3^; smoothing with an 8 mm FWHM Gaussian kernel; and detrending according to Macey et al. (2004).

### FMRI data analysis and statistical assessment

#### Univariate analysis

Using SPM12, a first-level model was constructed, incorporating HRF-convolved regressors for each of the following six conditions: *StimPress*, *StimFlutt*, *StimVibro*, *ImagPress*, *ImagFlutt*, and *ImagVibro*. For the Imagery conditions, the regressors were modeled using only trials rated as successful. Therefore, six participants had to be excluded because of empty regressors in one or more runs. Please note that all participants had almost 100% performance in the stimulation condition, therefore no additional modeling of unsuccessful stimulation was necessary. To allow for a later conjunction analysis, Null events were randomly assigned to and modeled in two separate regressors. Three additional regressors of no interest were included, as follows: (1) the precue, (2) the remaining (unsuccessful) imagery trials, and (3) the button presses. Modeling the precue for every trial as a regressor of no interest was done to account for the influences of spatial attention, which could potentially drive activation during mental imagery. On the subject level (first-level analysis), contrasts against implicit baseline were calculated for the six experimental conditions and the two groups of Null events, which were then forwarded to group-level (second-level) analyses, modeled with flexible factorial design specification of SPM.

To test for activations associated with perceptual or mental imagery processing, we first contrasted the mean of the three Stimulation and the three Imagery conditions, respectively, against Null events. Furthermore, we computed a conjunction against the conjunction null hypothesis (Friston et al., 2005), conjoining the contrasts *Stimulation > Null* and *Imagery > Null* using the estimates of the two independent Null event regressors. To establish cytoarchitectonic references, we used the Anatomy Toolbox version 2.2 (Eickhoff et al., 2005).

Next, to test for differences in perceptual processing of the three stimulus types, we computed second-level contrasts for the six possible pairings of the three Stimulation conditions (*StimPress*, *StimFlutt*, *StimVibro*). The same was done for the three Imagery conditions (*ImagPress*, *ImagFlutt*, *ImagVibro*).

#### ROI definition

To examine content-related activity within the somatosensory processing stream, we defined regions of interest (ROIs) based on the probabilistic cytoarchitectonic maps of the Anatomy Toolbox version 2.2 for contralateral S1 (BA3b, BA1, BA2) and bilateral S2 (OP1, OP2, OP3, OP4), with a 50% probability cutoff. To increase the specificity of our analysis to those aspects of S1 and S2, which are activated by our task, we intersected the anatomic masks with activation clusters resulting from the contrast *Stimulation > Null*. As univariate effects within the somatosensory processing stream only occurred in the three subregions of contralateral S1 as well as bilateral S2, we focused on these five resulting ROIs. For the visualization of ROIs, we used MRIcron version 2.1 (Rorden and Brett, 2000). Within these masks, we performed a decoding analysis to test for content-specific (Press, Flutt, Vibro) multivariate activation patterns.

#### Multivariate pattern analysis

To identify content-specific differences between the experimental conditions and to thus go beyond mere univariate BOLD activation differences, we used MVPA. All decoding analyses were performed using The Decoding Toolbox (Hebart et al., 2015), which allows the application of support vector machine (SVM) classification to neuroimaging data.

Except for smoothing and detrending, the same preprocessing steps as for the univariate analysis were applied to the data. For the decoding analysis, we used condition-specific β estimates from a first-level model with the same design as used for the univariate analyses. We performed two decoding analyses, where β estimates of the six conditions (*StimPress*, *StimFlutt*, *StimVibro*, *ImagPress*, *ImagFlutt*, and *ImagVibro*) were extracted (1) from the cortical clusters resulting from the (univariate) conjunction analysis, and (2) from the predefined ROIs. The β estimates were *z*-scaled (normalized) across the samples for each voxel to control for univariate effects and entered into the cross-validated classification schema. We used linear multiclass SVMs (libsvm) in a leave-one-run-out sixfold cross-validation scheme over the six runs, with the SVM trained on each possible selection of data from five runs and then tested on the data from the remaining sixth run. First, SVMs were trained and tested within the Stimulation and Imagination conditions, respectively (i.e., one decoding schema for *StimPress, StimFlutt* and *StimVibro,* and one decoding schema for *ImagPress, ImagFlutt* and *ImagVibro*). For a comprehensive display of the findings, we report results as “above chance decoding accuracy.” For all analyses, the chance level of the three-class SVM classification was 33.33%. Options for hyperparameter tuning as well as for feature and parameter selection were kept at default values, provided by the toolbox, to allow for comparisons with previous work and simplicity. The resulting confusion matrices of the different decoding analyses are provided in Extended Data Figure 4-1.

Next, we used the same leave-one-run-out sixfold cross-validation scheme to test for cross-classification (i.e., the classifier was trained on data from the Stimulation conditions and tested on the Imagery conditions, and vice versa). Results are presented analogously to the main decoding analysis.

As an additional control analysis, multiclass SVMs were trained and tested on all six conditions at once. For this decoding analysis, the chance level was 16.67%. As expected, the confusion matrices of the six-class decoding analyses show that Stimulation and Imagery conditions are clearly distinguishable (Extended Data Fig. 4-2).

To validate our results from the decoding analyses and account for potential non-normally distributed decoding accuracy values (Stelzer et al., 2013), we performed nonparametric permutation tests, as implemented in The Decoding Toolbox (Hebart et al., 2015), with 1000 iterations per ROI. For this purpose, a random permutation of the six labels (*StimPress*, *StimFlutt*, *StimVibro*, *ImagPress*, *ImagFlutt*, *ImagVibro*) was drawn for each fold of the leave-one-out cross-validation, keeping the classification parameters as default. After averaging over subjects, this resulted in five distributions (one for each ROI) of decoding-accuracy values with 1000 data points per ROI. These distributions were then used to calculate the probability of the occurrence of values of interest resulting from our decoding analysis, described above.

This was done by dividing the number of results for the ROI-matching reference set, which were of greater or equal value to the averaged results in question by the number of reference results of smaller value. The minimal possible *p* value was therefore approximately *p* = 0.001. This resulted in a *p*-value per ROI for Stimulation, Imagery, and cross-classification.

### Data availability

The code to run the experiment and analyze the data can be found on GitHub (https://github.com/Neurocomputation-and-Neuroimaging-Unit/Tactile_Imagery_Content_in_SI). The fMRI and behavioral data can be shared by the corresponding author on request.

## Results

### Behavioral data

**Figure 2.**
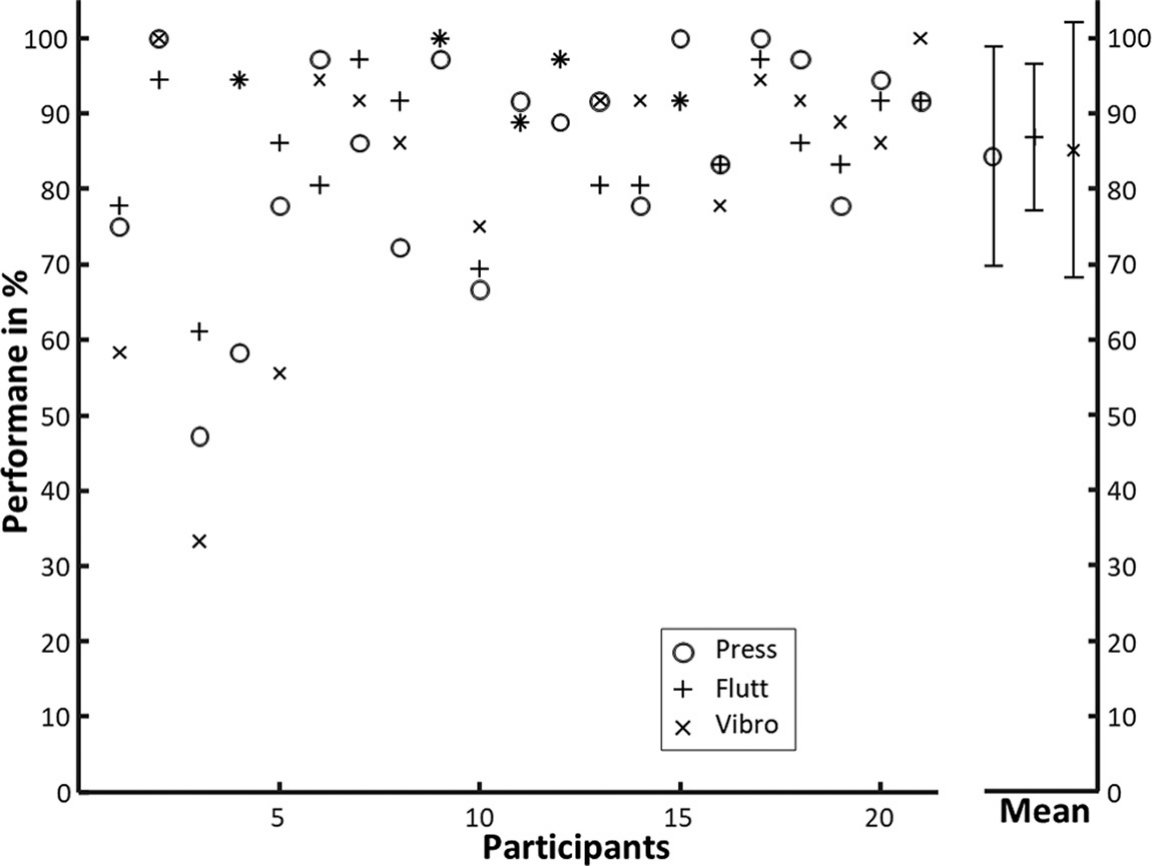
Performance in the Imagery conditions per stimulus type for the 21 participants included in the fMRI analysis. Means and SDs over all *N* = 21 are plotted in the right panel.

The response data of the *N* = 21 participants that were included for data analysis revealed a homogeneous performance regarding the Stimulation trials: 94.1% of the No-Stimulation trials were identified correctly (SEM =* *0.02); and 97.1% of the Stimulation trials were reported as perceived (SEM =* *0.01). The remaining 2.9% of responses to Stimulation trials were missed (SEM = 0.01). Figure 2 shows the performance (i.e., the percentage of trials rated as successful) in Imagery trials. No significant differences in Imagery success among the three types of to-be-imagined stimulations was found, when tested with a repeated-measures 1 × 3 ANOVA (*F*_(2,377)_ = 0.59, *p *=* *0.557).

To check for increasing or declining performance throughout the experiment (e.g., because of learning or tiredness), we computed a repeated-measures ANOVA over the three stimulus types with the six runs as a measure of time (3 × 6 factors). No significant main effect was found for the runs (*F*_(5,300)_ = 0.66, *p *=* *0.650) or interaction with stimulus type (*F*_(10,300)_ = 0.51, *p *=* *0.879).

The VTIQ was descriptively evaluated: 3.50 ± 0.59 (mean ± SD). For comparison, Campos and Pérez-Fabello (2009) report mean VVIQ-2 scores of 3.8 ± 0.6. The results of the VTIQ are therefore within an expected range compared with other tools assessing mental imagery.

To test whether VTIQ scores relate to the Imagery success in our paradigm, we correlated the VTIQ scores with participants mean success ratings. We found a significant correlation (*r *=* *0.74, *p *<* *0.001), indicating that the subjects’ self-assessment of their imaginative ability (VTIQ ratings) directly relates to the imagery performance report in our experimental paradigm.

### fMRI data

#### Univariate analysis of stimulation and imagery

**Figure 3.**
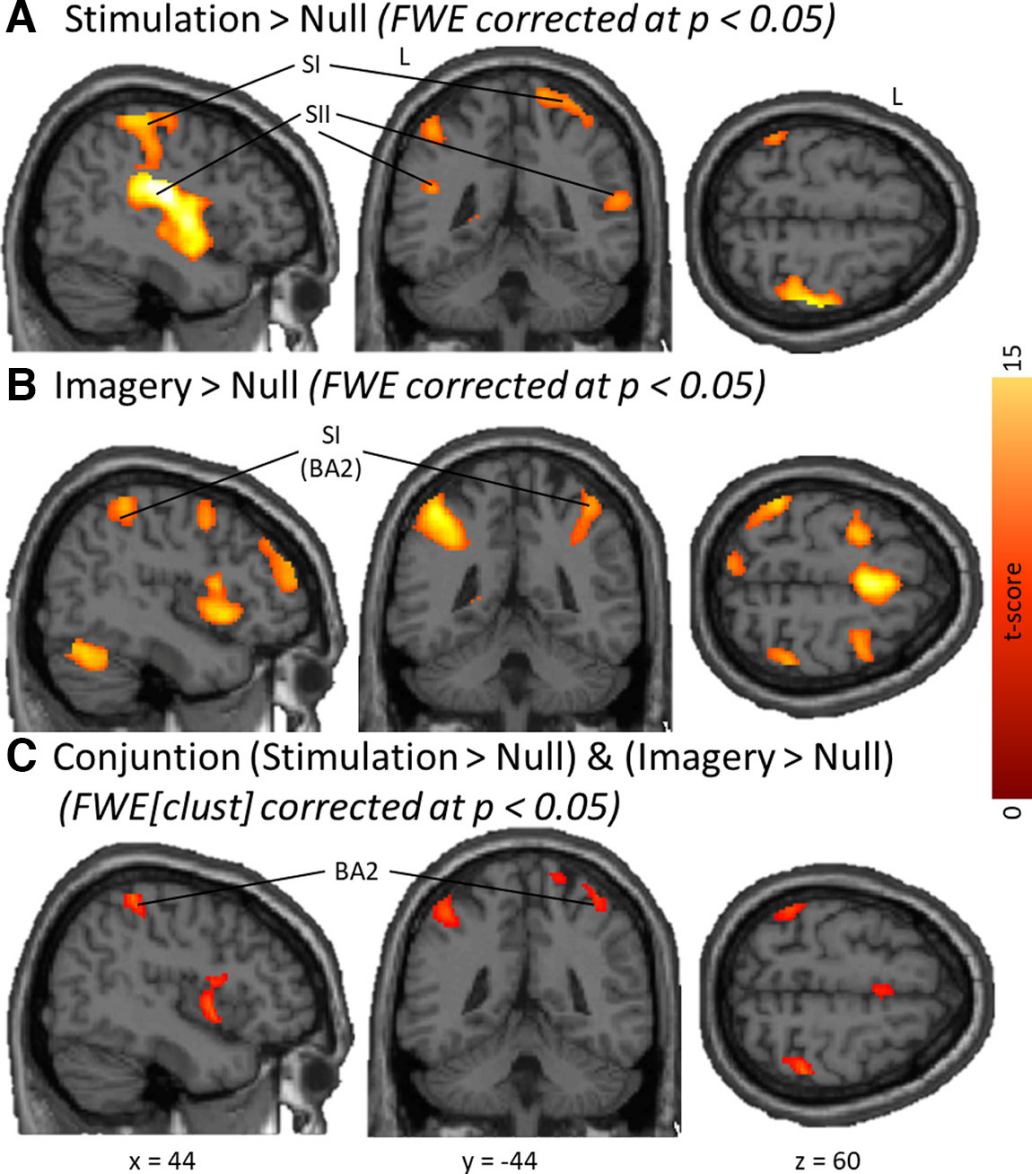
Results of univariate contrasts computed for the mean activation across the three stimulus types (Press, Flutt, Vibro). ***A***, *Stimulation > Null*, and ***B***, *Imagery > Null* are both reported at *p* < 0.05, whole-brain FWE corrected. ***C***, The Conjunction of the contrasts (*Stimulation > Null*) and (*Imagery > Null*) was FWE corrected at cluster level (*p* < 0.05).

To locate sensory areas activated by the applied vibrotactile stimulation, we first averaged the three stimulation conditions (*StimPress*, *StimFlutt*, *StimVibro*) and computed the contrast *Stimulation > Null* at *p* < 0.05 FWE corrected (Fig. 3*A*). We found strong activation in the right (contralateral to stimulation) S1 and bilateral S2. Additional clusters had their peak voxel in the parietal and frontal cortices, as well as left temporal areas (Table 1, complete report).

**Table 1:** Activation clusters (cluster size, >100 voxels) of the mean across the three different stimulus types (Press, Flutt, Vibro) for the different contrasts computed in the univariate fMRI data analysis

Anatomical region (peak)	Cluster size	MNI coordinates (peak)			*t* Score (peak)	*z* Score (peak)
		*x*	*y*	*z*		
Contrast *Stimulation > Null* (whole-brain FWE corrected, *p* < 0.05)						
R S2	7117	54	−16	20	09.81	Inf
Extending to R S1		54	−20	48	16.78	Inf
L S2	3495	−62	−28	22	13.54	Inf
L IPL	208	−44	−46	56	7.76	7.13
SMA	168	0	0	70	7.23	6.86
L cerebellum	106	−48	−54	−20	7.14	6.64
L cerebellum	207	−14	−72	−46	9.45	Inf
R inferior temporal	157	54	−58	0	8.68	7.83
Contrast *Imagery > Null* (whole-brain FWE corrected, *p* < 0.05)						
SMA	3457	0	2	70	14.25	Inf
R IFG	3350	54	12	0	13.93	Inf
L IFG	2633	−46	14	−4	12.10	Inf
L IPS	1477	−34	−48	44	11.63	Inf
L middle FG	1402	−44	30	26	9.77	Inf
L cerebellum	893	−40	−64	−24	11.18	Inf
R cerebellum	857	40	−54	−26	9.00	Inf
L precuneus	759	−6	−72	46	8.67	7.83
R middle FG	674	40	50	24	8.22	7.75
R BA 2	451	44	−42	58	8.00	7.33
R cerebellum	224	36	−62	−50	8.83	Inf
L IPL	172	−66	−28	28	6.39	6.02
Conjunction *Stimulation > Null* and *Imagery > Null* (FWE cluster-corrected, *p* < 0.05)						
R insula	962	60	10	−2	6.10	5.78
L IPL	817	−44	−46	56	5.68	5.41
R BA2	634	44	−40	60	6.06	5.74
L temporal pole	476	−58	6	2	6.44	6.07
SMA	323	0	2	70	5.78	5.50
L cerebellum	207	−48	−54	−20	5.35	5.13

R, Right hemisphere; L, left hemisphere; FG, frontal gyrus, Inf, infinite.

To identify brain areas activated by tactile mental imagery, we averaged the three imagery conditions (*ImagPress*, *ImagFlutt*, *ImagVibro*) and computed the contrast *Imagery > Null* at *p* < 0.05 FWE corrected (Fig. 3*B*). This showed activation of the posterior medial frontal cortex including the SMA and various parietal areas (Table 1). Notably, this contrast revealed an activation cluster with maximum in right BA2, according to the Anatomy Toolbox (Eickhoff et al., 2005).

To test for activation shared by Stimulation and Imagery, we computed the conjunction of the contrasts *Stimulation > Null* and *Imagery > Null*, presented FWE cluster corrected at *p* < 0.05 in Figure 3*C*. Shared activations can be found in SMA, right insula, left IPL, as well as bilateral temporal areas (Table 1). In line with the previous contrasts, this analysis confirms that the same subregion in right BA2 is activated during Stimulation and Imagery. When intersecting the conjunction with the somatosensory probabilistic cytoarchitectonic maps, 114 voxels are found in right BA2, but no activation in other ipsilateral or contralateral somatosensory cortices.

To test whether a univariate difference between the three stimulus types (Press, Flutter, Vibro) can be found in the somatosensory cortex, we investigated the BOLD activation within the somatosensory probabilistic cytoarchitectonic maps for the contrasts of the three Stimulation conditions (*StimPress*, *StimFlutt*, *StimVibro*) against each other. None of the six contrasts of pairs of conditions revealed any significant differences at *p* < 0.05 (FWE corrected). The only contrast showing any activation differences within the somatosensory processing stream is the contrast *StimVibro* > *StimPress*, where two clusters of six voxels each can be found in contralateral S1 and S2, respectively [S1 peak: *x* = 44, *y* = −20, *z* = 54, *t* score = 3.71, *z* score = 3.63, *p* = 0.998 (FWE cluster corrected); S2 peak: *x* = 38, *y* = −30, *z* = 20, *t*-score = 3.73, *z* score = 3.65, *p* = 0.998 (FWE cluster corrected)]. Computing the same contrasts between stimulus types within the Imagery conditions (*ImagPress*, *ImagFlutt*, *ImagVibro*) revealed no significant activation differences.

#### Multivariate analysis of stimulation and imagery

Using MVPA, we tested whether activation patterns allow distinguishing between stimulus types (i.e., the vibration frequency of the stimulation as well as its respective mental imagery content). When performing multiclass decoding analyses using the five cortical clusters resulting from the conjunction analysis as regions of interest, we find that the Stimulation conditions can be distinguished in all five areas, demonstrated by significant decoding accuracy minus chance values (mean ± SEM; right BA2: 10.8 ± 2.4%, *p* = 0.001; left IPL: 11.4 ± 2.7%, *p* = 0.001; SMA: 7.7 ± 3.4%, *p* = 0.001; right insula: 4.5 ± 2.9%, *p* = 0.002; left temporal pole: 5.8 ± 2.3%, *p* = 0.001). In contrast, the decoding accuracy for the Imagery conditions was, as expected, considerably lower and only significantly above chance in the following three clusters: right BA2 (2.7 ± 3.0%, *p* = 0.031), left IPL (2.4 ± 2.7%, *p* = 0.047), and SMA (4.2 ± 2.3%, *p* = 0.003). Finally, for the cross-classification trained on the Stimulation conditions and tested on the Imagery conditions, only the following two clusters showed significant results: the right BA2 (4.2 ± 1.8%, *p* = 0.001) and left IPL (5.0 ± 2.2%, *p* = 0.001). Cross-classification trained on Imagery and tested on Stimulation conditions showed no significant results.

**Figure 4.**
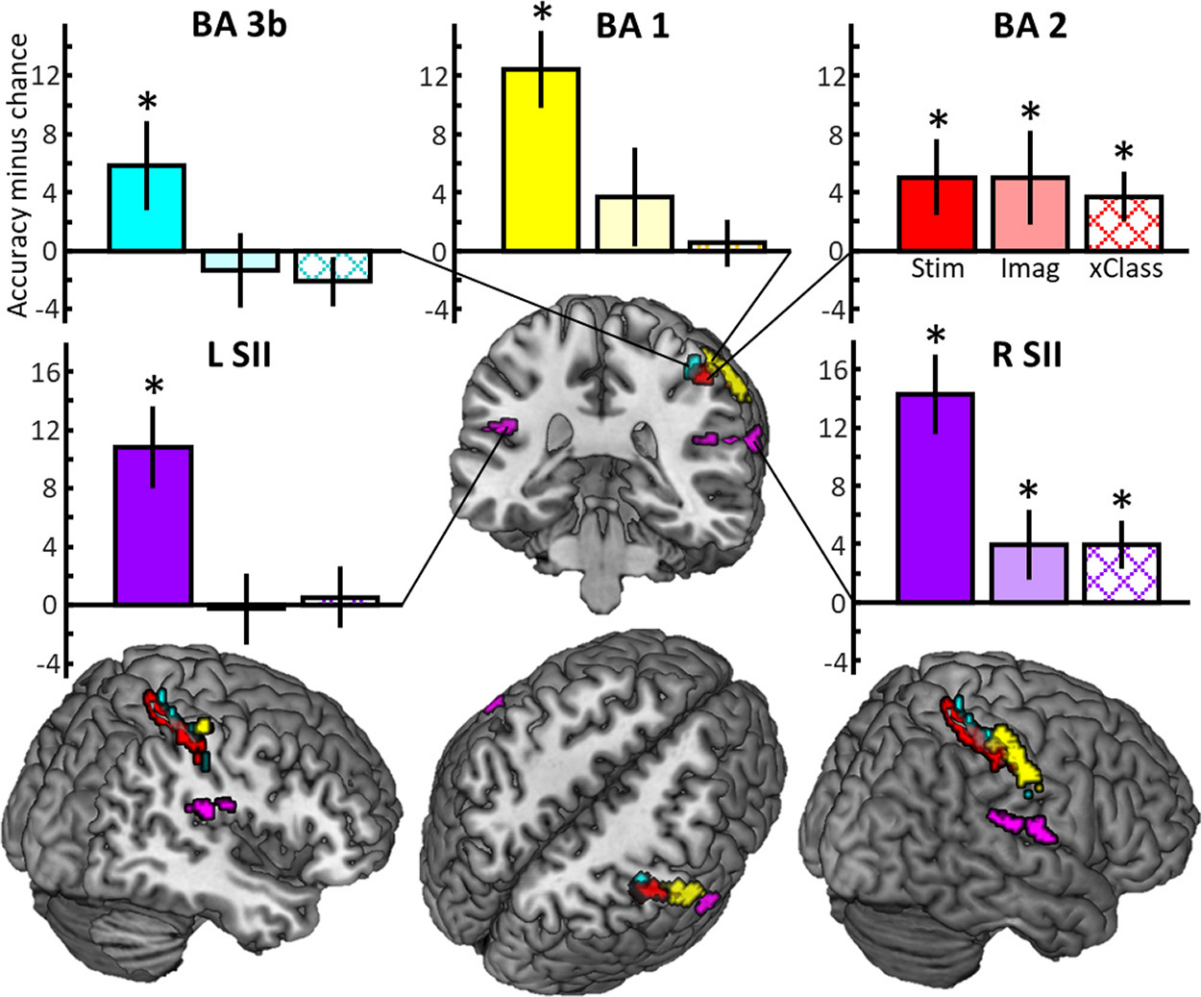
ROI-based MVPA and the resulting decoding accuracies minus chance. Decoding accuracies minus chance are presented as the mean across subjects of the decoding analyses for the three Stimulation conditions and the three Imagery conditions as well as the cross-classification (xClass) trained on the Stimulation and tested on the Imagery conditions. Error bars represent the SEM; asterisk marks significant decoding results at *p* < 0.05).

To further examine content-related activity patterns within the somatosensory processing stream, we used ROIs in contralateral S1 (BA3b, 60 voxels; BA1, 105 voxels; BA2, 157 voxels) and bilateral S2 (left S2, 181 voxels; right S2, 197 voxels), as derived from the intersection of the stimulation versus baseline contrast and anatomic masks from the Anatomy Toolbox (see Materials and Methods). For each ROI, the mean decoding accuracy minus chance values for Stimulation and Imagery are shown in Figure 4*A*. The multiclass decoding analyses within the Stimulation conditions revealed significant decoding results in all ROIs. When testing whether different imagery content leads to distinguishable activation patterns, we found significant decoding only in S1 subarea BA2 and contralateral S2. The cross-classification decoding analysis trained on the Stimulation conditions and tested on the Imagery conditions revealed accuracies significantly greater than chance in the same two ROIs. Corresponding confusion matrices can be found in Extended Data Figure 4-1.

10.1523/ENEURO.0408-22.2023.f4-1 Extended Data Figure 4-1 Confusion Matrices for multi-class decoding with three classes for each ROI, separate for the conditions Stimulation and Imagination, as well as for the cross-classification (xClass) trained on Stimulation and tested on Imagination. The percentage of assignment from each class to all classes is colour coded. Download Figure 4-1, DOCX file.

10.1523/ENEURO.0408-22.2023.f4-2 Extended Data Figure 4-2 Confusion Matrices for multi-class decoding with six classes for each ROI. The percentage of assignment from each class to all classes is colour coded. In general, fewer data is confused between conditions (Imagination vs. Stimulation), i.e. most classification errors are made between content of one condition. Download Figure 4-2, DOCX file.

## Discussion

This study addressed whether primary somatosensory cortices contribute to the representation of mental content during tactile imagery. In our fMRI paradigm participants either perceived or imagined three different vibrotactile stimulus types (Press, Flutter, Vibro). With conventional univariate analyses, we observe that tactile perception and imagery share activation in contralateral BA2, the hierarchically highest subarea of S1, thus confirming previous findings (Schmidt and Blankenburg, 2019), but it was not possible to distinguish the different imagery conditions. Using MVPA, however, we were able to differentiate the resulting patterns of somatosensory cortical activation: for the Stimulation conditions within all subregions of the somatosensory processing stream, whereas for the Imagery conditions most consistently within contralateral BA2. Furthermore, a cross-classification with training on Stimulation and testing on Imagery conditions was possible. Thus, we were able to show that the BA2 activation induced by Imagery reflects tactile mental content, where a similar neuronal activation pattern as that during the perception of corresponding stimuli was found.

### Perception and imagery of somatosensory stimuli

Both perception and mental imagery of vibrotactile stimuli showed expected fMRI activation in the univariate BOLD analysis. The processing of vibrotactile stimulation induces activation in contralateral S1 and bilateral S2, the typical evoked response for somatosensory stimulation (Francis et al., 2000; Nierhaus et al., 2009; Grund et al., 2021; Schröder et al., 2021). Tactile mental imagery activates a broad network of frontal and parietal regions, including the SMA, IFG, IPL, and intraparietal sulcus (IPS). This activation pattern, similar to the task-positive network, seems to be related to the general mental construction process, as it is activated irrespective of the mental content of imagery (Hassabis and Maguire, 2009; Zvyagintsev et al., 2013; Schmidt and Blankenburg, 2019). We additionally found an activation of the contralateral S1 during tactile mental imagery. The overlap of S1 activation revealed by the conjunction of *Stimulation > Null* and *Imagery > Null* emphasizes that perceptually activated areas are also recruited during mental imagery. According to the “sensory recruitment theory,” early sensory areas contribute to the mental representation of a sensory stimulation during mental imagery (Kosslyn et al., 2001; Pasternak and Greenlee, 2005; Serences, 2016). Our finding of activation in S1 during imagery replicates the results of Schmidt and Blankenburg (2019), where, similar to the study at hand, mental imagery of simple vibrotactile stimuli only activated the hierarchically highest S1 subarea BA2. This finding can be interpreted as further evidence for sensory recruitment where only very fine-grained, detailed mental imagery of high vividness activates lower-order sensory areas (Kosslyn and Thompson, 2003; Schmidt et al., 2014). In the somatosensory system, this was shown with mental imagery of more complex spatial information with cortical activation down to BA1 (Schmidt et al., 2014). In contrast, the mental imagery of abstract concepts or symbolic, language-based content does not require the recruitment of regions that are specialized in the processing of perceptual stimulus features (Lee et al., 2013; Lee and Baker, 2016; Christophel et al., 2017). In brief, our results so far show that tactile mental imagery (1) activates frontoparietal areas, whose general contribution to mental processes is well described; and (2) goes along with sensory recruitment of BA2 within S1.

### Content-specific activation in primary somatosensory cortex

But does the observed activation of BA2 also relate to the representation of a specific mental content? While the presentation of different stimulus types led only to subtle activation differences for the *StimVibro* > *StimPress* contrast, univariate analyses did not reveal any activation differences during respective mental imagery. The applied types of vibrotactile stimulation (different content) were designed to all comprise 2 Hz half-sine wave pulses. For one stimulation type, the 2 Hz pulses were unmodulated (Press), the other two stimulation types were modulated by 30 or 150 Hz, corresponding to the Flutter and Vibration frequency range, respectively. Thus, we tried to minimize the physical and perceptual difference between the three different stimulus types and focus the participant’s perception on the applied frequency (Press, Flutter, Vibro). The perception of these different vibrotactile frequencies have been linked to different mechanoreceptors: slowly adapting Merkel cells sensitive to frequencies <10 Hz, rapidly adapting Meissner’s corpuscles responding most strongly to frequencies in the flutter range, and Pacinian corpuscles most sensitive to high-frequency vibrations >100 Hz (Harrington and Hunter Downs, 2001; Friedman et al., 2004). Vibrotactile stimulation in these different frequency ranges have previously been shown to elicit neural responses differentiable with noninvasive neuroimaging. Using EEG, evoked responses in S1 were found for 24 Hz stimulation, while 240 Hz stimulation evoked responses in both S1 and S2 (Hämäläinen et al., 1990). In an fMRI experiment, 150 Hz compared with 35 Hz stimulation showed stronger activation in posterior parietal cortex (PPC) and S2 (Harrington and Hunter Downs, 2001). Furthermore, using fMRI, the contralateral S1 was also shown to carry frequency-dependent information (Kim et al., 2016). Therefore, our finding, pointing toward a slightly stronger activation in contralateral S1 and S2 for the *StimVibro* condition, is well in line with the literature.

During mental tactile imagery, we observe activation in contralateral S1 (BA2), which is suggestive of content-specific activation, in the sense of sensory recruitment theory. However, we did not find univariate activation differences among the three Imagery conditions, namely the three types of mental contents. We used multivariate decoding analysis, as a more sensitive test for differences in the local voxel activation pattern. First, we tested for the possibility to classify the different stimulus types within the regions revealed by the conjunction analysis between Stimulation and Imagery condition. It was possible to classify the three different types of vibrotactile stimulation in all five regions, whereas the corresponding three Imagery types were decodable only from the SMA, left IPL, as well as right BA2 clusters. Strikingly, cross-classification, where the classifier was trained on the Stimulation and tested on the Imagery conditions, was possible only in left IPL and right BA2. These findings show content-specific activation during imagery within S1, in line with sensory recruitment theory. The fact that our analysis also showed sensitivity to the mental content in IPL and SMA might be related to content-specific contributions of the mental construction process, or alternatively, differences in the cognitive strategy to imagine the different stimulus types.

Building on this finding, we next wanted to test whether content-specific codes are specific to BA2 and whether other regions within the somatosensory processing stream also exhibit content-specific codes during mental imagery. To this end, we used ROIs derived from the Anatomy Toolbox, but limited to areas that were generally activated for Stimulation. Thus, we increased the specificity of our decoding results to those areas and subareas within S1/S2, which were activated by the vibrotactile stimulation. We found that for Stimulation, the three stimulation types can be classified throughout the somatosensory processing stream. Most interestingly, the three Imagery conditions can only be classified in contralateral BA2, the hierarchically highest subarea in S1, as well as in contralateral S2. Furthermore, above chance cross-classification was also possible in contralateral BA2 and S2. Therefore, our MVPA revealed content-specific activation during mental tactile imagery within the contralateral S1, which relies on similar patterns as activated by stimulation.

The neuronal foundations that underlie the differences in local activation patterns, however, remain speculative. Previous studies that decoded from S1 used stimuli with differentiable spatial layout (Schmidt et al., 2014); therefore, decodability was most likely based on fine-grained somatotopic organization. In the study at hand, spatial features were kept constant, but the stimuli were designed so as to activate different mechanoreceptors in the skin. Although it seems unlikely that either the different stimuli activate one receptor exclusively or that signals from different receptors activate strictly distinct neuronal populations in S1 (Saal et al., 2015), an activation of overall different patterns of neuronal populations can still be expected. However, since the spatial distribution of such populations is below the resolution of MRI, decoding is potentially based on hyperacuity (Boynton, 2005; Kriegeskorte et al., 2010), which again needs to be investigated in future studies with higher resolution.

Together, our results for the first time provide direct evidence that S1 activation during mental imagery is specific to a mental content. Moreover, the fact that mental content is represented in BA2 but not in hierarchically lower subregions of S1 supports the hypothesis of top-down sensory recruitment.

### Mental imagery and working memory

As both mental imagery and working memory (WM) rely on internal mental representations, sensory recruitment is also discussed in the context of sensory working memory studies (Tong, 2013; Christophel et al., 2017). The main difference between the two paradigms lies in the proposed way of how a mental image comes into being: while the internal representations created in working memory tasks result from an externally initiated process, mental imagery is assumed to internally activate mental representations through a top-down driven processing (Mechelli et al., 2004; Stokes et al., 2009; Dijkstra et al., 2017). The evidence for sensory recruitment is mixed across WM studies (Serences, 2016; Christophel et al., 2017; Uluç et al., 2018; Wu et al., 2018), similar to what has been reported for mental imagery studies (Kosslyn and Thompson, 2003). For the visual modality, working memory and mental imagery have previously been found to rely on similar primary visual areas (Albers et al., 2013; Tong, 2013). Also, a series of tactile working memory decoding studies tested the cortices that retain information during delay phases. Schmidt et al. (2017) have recently shown that the frequency of a vibrotactile stimulus can be decoded from activation in right IFG, while information on the spatial layout of tactile stimuli can be decoded from the PPC (Schmidt and Blankenburg, 2018). Interestingly, Schmidt and Blankenburg (2018) found content-specific activity in S1 only in an early phase of a 12 s WM retention period. This implies that in WM the tactospatial information is initially represented in S1, but with a longer retention—and potentially a less vivid mental representation—it is rather hierarchically higher-order areas that exhibit content-specific activation. One interpretation of these findings further supports the idea, discussed above, that the extent of activation in S1 corresponds to the vividness of tactile mental imagery (Kosslyn and Thompson, 2003), while the mental representation loses this phenomenal character with longer retention and the mental image becomes rather abstract in its nature.

### Mental imagery and attention

Another challenge for both working memory and mental imagery research, is to distinguish between attentional mechanisms and the representation of mental content. Conceptually, it is difficult to draw a clear-cut distinction between attentional mechanisms that support the maintenance of a specific mental content and the mental content representation as such (Schmidt et al., 2021). In our paradigm, we included No-Stimulation trials, where the participants were only directing their spatial attention to the stimulation site while they did not have to imagine a stimulus, nor was a stimulus applied. By modeling the precue for every trial as a regressor of no interest, we therefore tried to account for the influences of spatial attention concerning the time, place, and type of a stimulus, which might potentially drive activation during mental imagery. This makes it highly unlikely that our findings of activation during tactile mental imagery were driven by spatial attention. The conjoined activations we found for Stimulation and Imagery depict major components of the commonly reported task-positive network elicited by tactile imagery. This is in line with previous studies on auditory and visual mental imagery, which found activation in multimodal and various frontal areas, which are also associated with attentional processes (Zvyagintsev et al., 2013; Lückmann et al., 2014). Furthermore, a model containing neural correlates of selective internal attention during the prioritization of information in working memory tasks attributes activity in the pre-SMA, frontal-eye field, IPS and IPL to attentional processes (Myers et al., 2017), which is in line with our findings and with the suggestion that working memory and mental imagery share core processes (Tong, 2013). Finally, our finding that tactile imagery content can also be classified in IPL fits with the reported role of the parietal cortex in tactile attention (Goltz et al., 2015), suggesting that attentional mechanisms appear to be interwoven with the activation of a specific mental content.

### Conclusion

We show that mental content representations of vibrotactile stimuli can be found in a specific activation pattern within the hierarchically highest subregion of S1, namely BA2. We further found that the activation in sensory cortices induced by tactile imagery relies on patterns similar to those activated by tactile stimulation. Our results are in line with the view of mental imagery as an internally driven process involving frontoparietal areas as well as sensory recruitment of primary somatosensory cortex.

## Synthesis

Reviewing Editor: Anne Keitel, University of Dundee Decisions are customarily a result of the Reviewing Editor and the peer reviewers coming together and discussing their recommendations until a consensus is reached. When revisions are invited, a fact-based synthesis statement explaining their decision and outlining what is needed to prepare a revision will be listed below. The following reviewer(s) agreed to reveal their identity: Tobias Heed, Luke Miller.

The reviewers and I agreed that this is an interesting and well-conducted study that addresses a novel question. Nonetheless, the reviewers had some questions and suggestions, mainly regarding methods and results. Most notably, reviewer #2 suggested re-running the MVPA, which will allow for some additional insights.

I am attaching the unabridged reviewer comments below, to allow a point-by-point reply.

Reviewer #1 - Advances the Field (Required)

answers a question that hasn’t been answered before.

Reviewer #1 - Comments to the Authors (Required)

In this study, human participants either received tactile stimulation, or imagined the same stimuli while their BOLD signals were recorded (fMRI). There were three different stimuli. All three stimuli led to comparably increased blood flow (“activation”) in S1, both during stimulation and imagery. However, activation patterns across voxels (MVPA) differed between the three stimuli, again both for stimuation and imagery. Moreover, the experiment included a control condition in which participants directed their attention to the location at which stimuli would occur, but were not stimulated. This attention control allows distinguishing imagery- from attention-based activation.

This is a sound study. I have no major criticism. I find the experimental paradigm clear and adequate. The paper is well-written and easy to read. I think that the study is well seuited for eNeuro. Below I will list some aspects that can be addressed to improve the paper. I judge none of them major.

Respectfully submitted, signed, Tobias Heed (@editor, please do not remove my name)

1, the manuscript contains neither page numbers nor line numbers. this makes communicating about the paper tedious. Please consider adding both to future manuscripts and to the revision.

I will refer to the page numbers of the downloaded document as they appear in a pdf reader.

2, abstract, the first sentence sounds as if normal touch does not activate S1.

3, abstract, contributing supporting cognitive functions to mental processes, to me sounds about as empty as one can get. What are these words supposed to mean? They add no useful information to me as a reader.

4, p5, internal process, aren’t all processes internal?

5, p5, supporting appears again, it would be helpful if the term was better defined. Do the authors assume that these are processes which “improve” imagery if they exist, but would not lead to imagery being impossible if they were not available? A few lines down, attention - first said to be “supporting” is now titled “a crucial part of mentail imagery”. I find that the ideas/concepts remain unclear. (nb, these things are discussed much more clearly in the discussion, but they do not become clear hear at the outset)

6, p5, subarea 2 is called the “hierarchically highest” after a sentence referring to S1 and SII. Probably this only refers to S1 though?

7, p6, the intro presents MVPA for touch imagery from 2 perspectives: technical and functional. This sounds as if, had the authors not found any effects, it would not be clear whether this was a matter of the decoder not being able to find existing differences, or such differences truly not existing. While this may, of course, be an issue, it doesn’t make for a very convincing intro to the study.

8, p6, end of intro, it remains unclear why attention is controlled for (as opposed to other potential factors), and it doesn’t link to what was said earlier in the intro about attention.

9, p12, what is the rationale to baseline correlations against Attention rather than Null? It would be interesting to see the corresponding Null-based correlation on p19.

10, p15, I am surprised that 58% of BA2 were active. Do I understand this correctly, that 58% of all BA2 along all of the S1 strip were modulated by Imagery? If so, then I would be curious to read about what the authors think is going on in BA2. Would one not rather have expected strongly confined activation around the finger/hand area? Would imagery be distinguishable for different imagined target regions on the hand (one would think so, I guess, but the wide-spread activation makes me wonder)? Related to this point, did the authors try to decode in subvolumes within their ROIs (i.e., a searchlight), or is BA2 too thin for this? ROI sizes are not specified in the paper, which makes it difficult to judge these kinds of questions.

11, p19, when the authors say “activation”, do they mean “decoding accuracy for activation patterns”? More generally, I am unclear about what effect sizes were calculated.

12, Fig 4, there is an effect size for attention, but no other bars for attention. It is unclear to me what effect size this is. The corresponding “raw” bars could be added to the other subplots (?)

13, p21ff, I would find it interesting to read about the authors’ thoughts about whether imagined stimuli would be separable also if the perception of their real counterparts did not rely on different receptor types. Here, a strict interpretation could be that “content” is restricted to imagining the input of particular stimulus classes, based on the sensors underyling their perception. But the claim of the paper is more general (or, at least this restriction is not discussed) and would include differences in S1 activation for 2 stimuli for which true perception is based on identical receptors. Yet, the discussion on working memory hints at the possiblity that such distinctions would maybe recruit different - more? hierarchically lower? - S1 regions.

discussion overall: could maybe be streamlined a little to make it a bit shorter.

typos/language

p14, “noticeable are some outliers” - weird (German) word order

p20, pattern => s missing

p25 => constructions => remove s

Reviewer #2 - Advances the Field (Required)

The present study is the first to my knowledge to show that S1 represents the contents of tactile imagery.

Reviewer #2 - Comments to the Authors (Required)

The present study used fMRI combined with MVPA to investigate whether primary somatosensory cortex represents the content of tactile mental imagery. The task included three conditions: stimulation, imagery, and an attentional control. In the stimulation condition, three types of stimuli (pressure, flutter, vibration) were administered to the left index finger. In the imagery condition, participants imagined feeling these different stimulations. The three types of stimulation could be decoded using an SVM in several early sensory regions (BA3b, BA1, BA2, and SII). Interestingly, the contents of imagery could be significantly decoded from SI but only from BA2, a higher-level subregion of SI. The level of activation was related to the self-rated vividness of the tactile imagery. The authors conclude that this is further evidence that sensory regions are involved in mental imagery.

This is a well-conducted study that addresses and interesting and timely question. The manuscript is well-written and easy to follow. I do have some concerns, both major and minor, that I detail below.

Major issues

1) I think a bit more caution is needed when framing the stimuli as activating different mechanoreceptors (e.g., in the Introduction). While it is true that flutter typically activates FA1 and high-frequency vibration FA2, this is not necessarily the case and depends on other features of the stimuli (e.g., stimulation strength). Furthermore, it is unlikely that there are distinct populations for each in S1 (see work by Hannes Saal and Sliman Bensmaia), though I’d agree that they likely lead to different patterns of population activity.

2) For the univariate analysis of content, what is the rationale for comparing one condition versus the mean of the other two? Instead of comparing to them each individually, which is done in the MVPA.

3) Some important methodological points on the SVM are missing, which should be added. Specifically, the authors should report which type of kernel was used and how the hyperparameters were tuned.

4) Relatedly, the chosen binary SVM approach is suboptimal. While it is true that SVM technically compares two classes, there are multiclass approaches that allow you to decode more than two at a time. I recommend redoing the MVPA analyses to simultaneously decode all three stimulation types from each other (33% is chance), instead of taking the average of the binary outputs (50% is chance). An additional benefit of this approach is the ability to construct confusion matrices, which give insight into the types of errors made by the classifier. For example, flutter and vibration may be more likely to be confused than pressure and vibration.

5) Finally, I think there are some additional questions that might be interesting to address and would be made possible by the above multiclass approach. For example, can a classifier decode all six types of content (three stim, three imagery) and where does it make most errors (content or condition)? Can a decoder be trained using the stimulation condition and then significantly decode the imagery condition (and vice versa)?

Minor issues

1) Can the authors explain a little more why stimuli were applied to the left index finger and not the right, which is the dominant for most of their participants. Would they expect there to be differences in stimulus processing and imagery for the non-dominant versus dominant hand?

2) The Attention control condition seems like it would also include violations of expectation (to feel a stimulation) and therefore brain activity may contain some prediction errors. I don’t think this would change how the condition is used, but perhaps worth discussing briefly?

3) Did the authors also look at the relationship between decoding performance and imagery strength?
