# Supplementary material for: Content Representation of Tactile Mental Imagery in Primary Somatosensory Cortex
Source: eNeuro. 2023 Jun 2;10(6):ENEURO.0408-22.2023. doi: 10.1523/ENEURO.0408-22.2023 (PMC10249945; doi:10.1523/ENEURO.0408-22.2023)
Supplement: Extended Data Figure 4-1 — Confusion Matrices for multi-class decoding with three classes for each ROI, separate for the conditions Stimulation and Imagination, as well as for the cross-classification (xClass) trained on Stimulation and tested on Imagination. The percentage of assignment from each class to all classes is colour coded. Download Figure 4-1, DOCX file. [file enu-eN-NWR-0408-22-s02.docx]

**Stimulation Imaginaton xClass**

**BA3b**


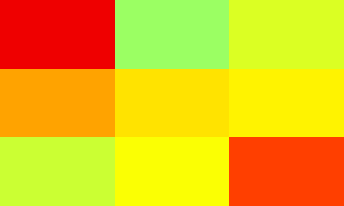


stim-press

stim-flutt

stim-vibro

**BA1**


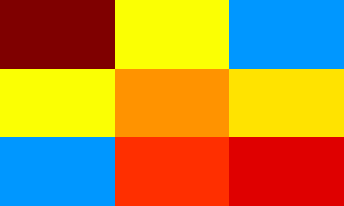


stim-press

stim-flutt

stim-vibro

**BA2**


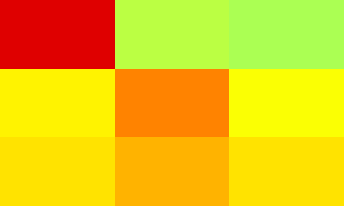


stim-press

stim-flutt

stim-vibro

**SII le**


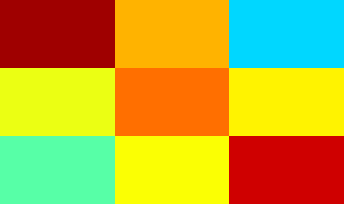


stim-press

stim-flutt

stim-vibro

**SII ri**


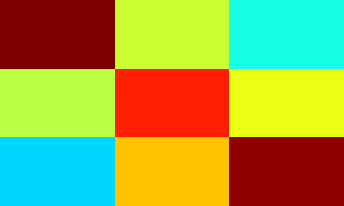


stim-press

stim-flutt

stim-vibro

stim-press

stim-flutt

stim-vibro

**BA3b**


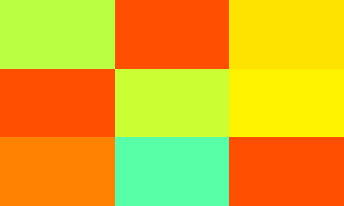


imag-press

imag-flutt

imag-vibro

**BA1**


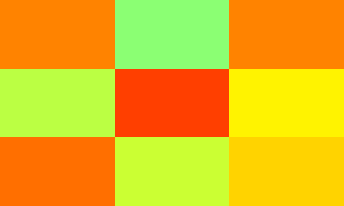


imag-press

imag-flutt

imag-vibro

**BA2**


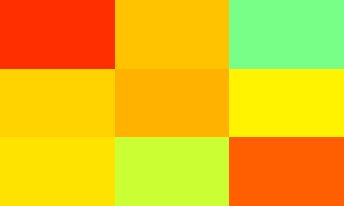


imag-press

imag-flutt

imag-vibro

**SII le**


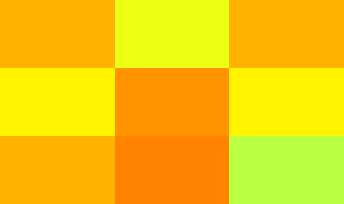


imag-press

imag-flutt

imag-vibro

**SII ri**


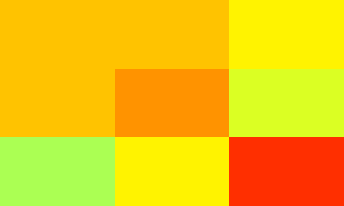


imag-press

imag-flutt

imag-vibro

imag-press

imag-flutt

imag-vibro

**BA3b**


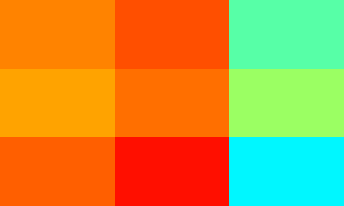


press

flutt

vibro

**BA1**


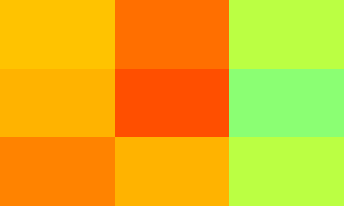


press

flutt

vibro

**BA2**


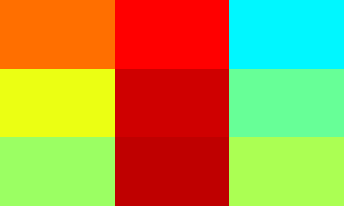


press

flutt

vibro

**SII le**


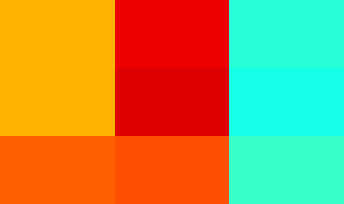


press

flutt

vibro

**SII ri**


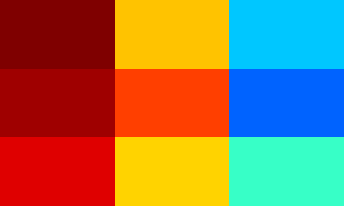


press

flutt

vibro

press

flutt

vibro


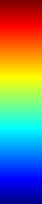


0

50%

**Figure 4-1**: Confusion Matrices for multi-class decoding with three classes for each ROI, separate for
the conditions Stimulation and Imagination, as well as for the cross-classification (xClass) trained on
Stimulation and tested on Imagination. The percentage of assignment from each class to all classes is
colour coded.

**BA3b**


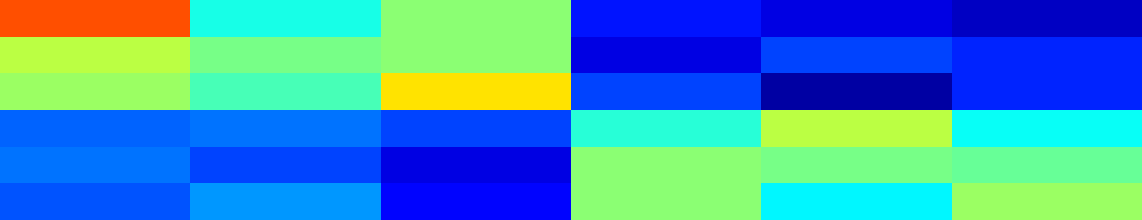


stim-press

stim-flutt

stim-vibro

imag-press

imag-flutt

imag-vibro

**BA1**


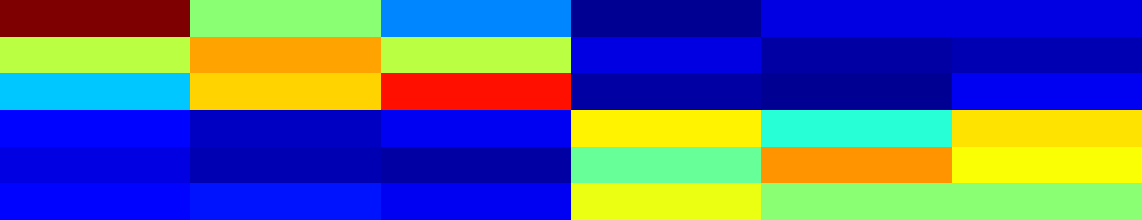


stim-press

stim-flutt

stim-vibro

imag-press

imag-flutt

imag-vibro

**BA2**


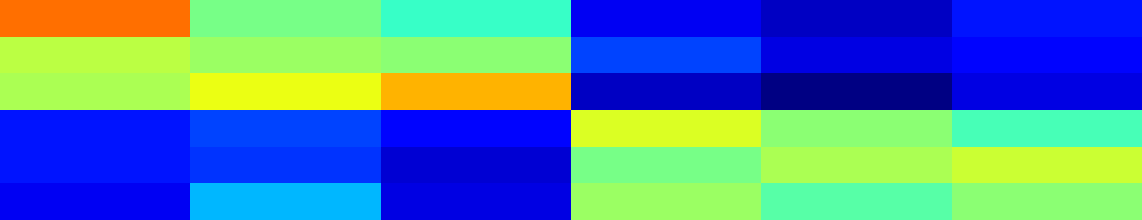


stim-press

stim-flutt

stim-vibro

imag-press

imag-flutt

imag-vibro

**SII le**


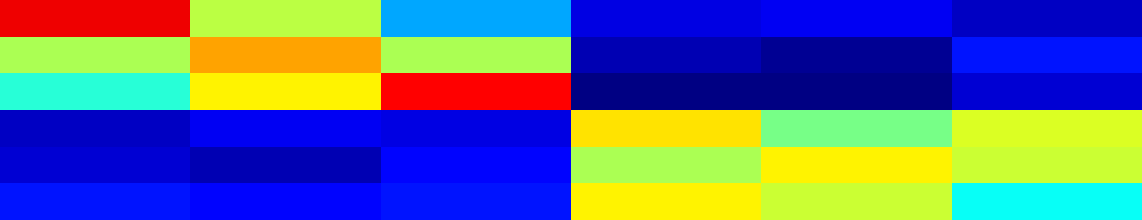


stim-press

stim-flutt

stim-vibro

imag-press

imag-flutt

imag-vibro

**SII ri**


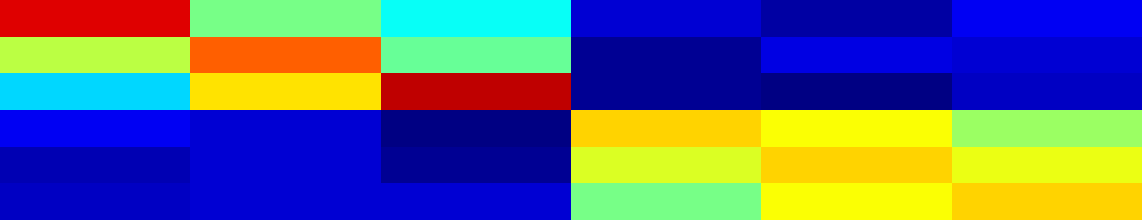


stim-press

stim-flutt

stim-vibro

imag-press

imag-flutt

imag-vibro

stim-press

stim-flutt

stim-vibro

imag-press

imag-flutt

imag-vibro


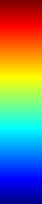


0

50%

**Figure 4-2**: Confusion Matrices for multi-class decoding with six classes for each ROI. The percentage of assignment from each class to all classes is colour coded. In general, fewer data is confused between conditions (Imagination vs. Stimulation), i.e. most classification errors are made between content of one condition.
